# Anchoring of a Kinked Uncemented Femoral Stem after Preparation with a Straight or a Kinked Reamer

**DOI:** 10.1111/os.12490

**Published:** 2019-07-22

**Authors:** Markus Heinecke, Frank Layher, Georg Matziolis

**Affiliations:** ^1^ Orthopaedic Department University Hospital Jena Campus Eisenberg Eisenberg Germany

**Keywords:** Kinked reaming, Kinked stem, Primary stability, Revision hip arthroplasty, Straight reaming

## Abstract

**Objective:**

To investigate a stem‐adjusted preparation of the femur with a kinked reamer and to determine whether this approach results in higher primary stability of a kinked stem than straight reaming of the intramedullary canal.

**Methods:**

Ten cementless stems with a kinked design were implanted in synthetic femurs after preparation of the femoral canal with 2 different reamer designs (straight reaming [SR] group *vs* kinked reaming [KR] group). The specimens were analyzed using CT to determine the anchoring length and examined with a mechanical testing system to establish their axial stiffness, torsional stiffness, and migration distance after 10 000 gait cycles.

**Results:**

The stem migration distances did not differ significantly between the groups (SR group 0.51 ± 0.16 mm *vs* KR group 0.36 ± 0.03 mm, *P* = 0.095). Only for the SR group, a correlation was found between the completely conical anchorage length and absolute stem migration (*P* < 0.05, *R* = 0.89). Regarding the torsional stiffness, no differences were observed between the study groups (SR group 6.48 ± 0.17 Nm/° *vs* KR group 6.52 ± 0.25 Nm/°, *P* = 0.398). In the KR group, significantly higher axial stiffness values were measured than in the SR group (SR group 1.68 ± 0.14 kN/mm *vs* KR group 2.09 ± 0.13 kN/mm, *P* = 0.008).

**Conclusions:**

The implantation of a kinked stem after kinked conical intramedullary preparation of the proximal femur showed equivalent results regarding anchoring length, stem migration, and torsional stiffness to those for straight conical reaming. The specimens with kinked reaming showed significantly higher axial stiffness values.

## Introduction

Currently, implantation of cementless stems is the gold standard for revision and fracture arthroplasty in the proximal femur with the aims of long diaphyseal anchoring and long‐term stability of the prosthesis^1^. The establishment of a modular type of prosthesis for implantation has been shown to offer two advantages: a distal stem fixing component and a proximal component that maintains the correct leg length and adjusts antetorsion and offset. Because the osseous prosthesis bed is often enlarged, sclerosed and thin, the repeat revision rate after implantation of a cementless prosthesis is lower than that after implantation of a cemented prosthesis^2^, ^3^. For cementless prostheses, the press‐fit method has been deemed the best choice for obtaining an interface between the bone and the implant, with the aim of achieving good stability, resulting in micromotion of less than 50 μm per gait cycle for bony ingrowth^4^, ^5^, ^6^. In addition, the amount of pre‐tension resulting at this interface must be large enough to counteract any destabilizing axial and rotational forces. Utilization of a conically shaped stem is the best method to achieve sufficient pre‐tension. Using this approach, the distal circulated area of fixation of the implanted stem in the isthmus of the femur achieves conical–conical anchoring^7^. The forces that act vertically are transformed by the inclined plane into forces that press against the bone into the implant. Loads are evened out, which allows the stem to be wedged back into place, thus enabling a good stability, even after an early migration. An increase in implant osteointegration results in secondary stability of the revision prosthesis^8^, ^9^. However, primary stability can also be attained *via* multiple points impacting on the implant in the osseous medullary cavity. Nonetheless, if the prosthetic stem does migrate, it is much less likely that secondary fixation of the implant will be necessary.

Regarding the geometry of the prosthesis, implants have been developed with different biomechanical considerations for stable stem anchoring. One type of implant has curved or kinked prosthetic stems because these compensate for the need for an additional osteotomy in the femoral medullary cavity, which may be necessary during the implantation of a straight stem. Curved or kinked stems should follow the anatomical pattern of the femur better than straight stems so that more extensive anchorage is possible^10^. Regardless of the stem design, impaction involving a conical and 3‐point combination is inevitable, even subsequent to an optimal preparation. In this regard, the preparation of the intramedullary canal for a cementless revision stem warrants closer examination because many different processes may be applied.

For straight stems, the femoral bone canal is drilled by a reamer, matching the design of the stem. The implantation of a curved stem occurs after the femur is prepared by drilling with a straight or flexible reamer. The latter is determined by the given femoral geometry. Thereafter, accurate matching between the femur and an anatomic curved stem cannot be achieved. A compromise between the non‐anatomic straight stem and the anatomic curved stem is the kinked stem. This stem is also not anatomically shaped, but it follows the antecurvation or varus form of the femur better than a straight stem. In contrast to a curved stem, a kinked stem allows the customized preparation of the femur with a kinked reamer. The outcomes of femoral revision using cementless fluted modular stems have previously been published by several research groups, and these studies have demonstrated good results at short‐term and long‐term follow‐up evaluations^3^, ^11^, ^12^, ^13^. However, to our knowledge, only a few biomechanical studies have investigated the migration resistance and stiffness of uncemented stems in revision hip arthroplasties^14^, ^15^, ^16^, ^17^, ^18^. To date, no studies are available on the possible relevance of the proximal femoral intramedullary preparation.

Therefore, the objective of this study was to evaluate a stem‐adjusted preparation of the femur with a kinked reamer and to determine whether this approach results in greater primary stability of a kinked stem in contrast to the straight reaming of the intramedullary canal.

## Methods

### *General Testing Strategy*

**Figure 1:**
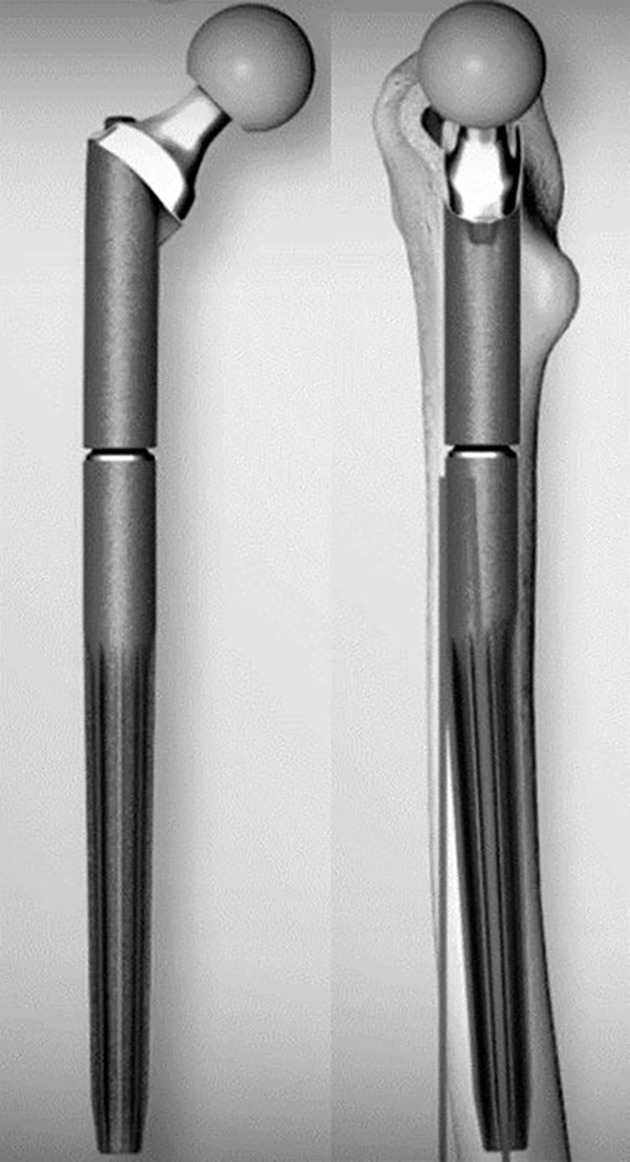
LINK© Kinked tapered (2°) proximal femoral stem with 3° antecurvation.

**Figure 2:**
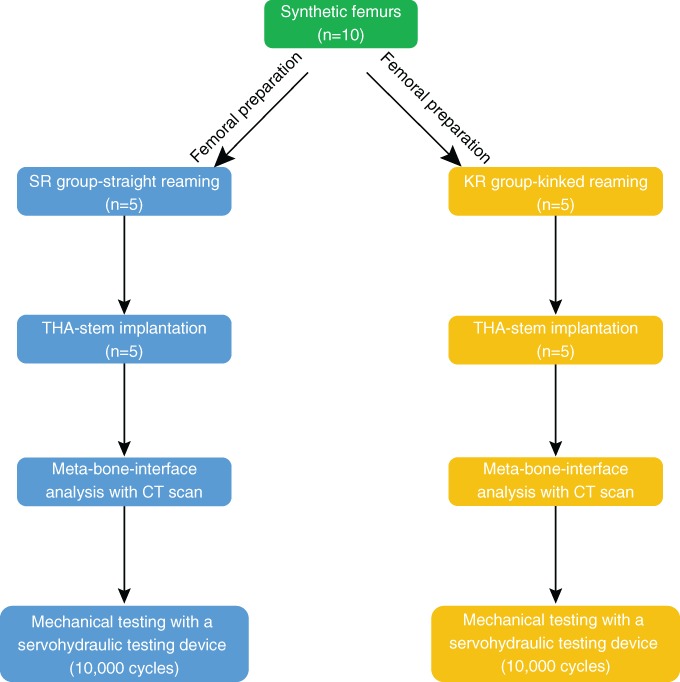
Flowchart of the general testing strategy.

For evaluation in this study, we selected a cementless stem (MP reconstruction prosthesis, LINK, Hamburg, Germany: a tapered [2°] fluted stem with a 3° angular bow, 14 mm diameter, 210 mm length, and 56 mm neck, Fig. 1) with a kinked design that had been used clinically for revision in total hip arthroplasty (THA). These stems were implanted in synthetic femurs after preparation of the femoral canal with two different reamer designs. The specimens were divided evenly into groups, with five specimens in each group. The implants were tested with respect to their axial/torsional stiffness and migration resistance (Fig. 2). The test guidelines and parameters used were the same as those described previously for the analysis of synthetic femoral bones using press‐fit stems^19^, ^20^.

### *Specimen Preparation*

**Figure 3:**
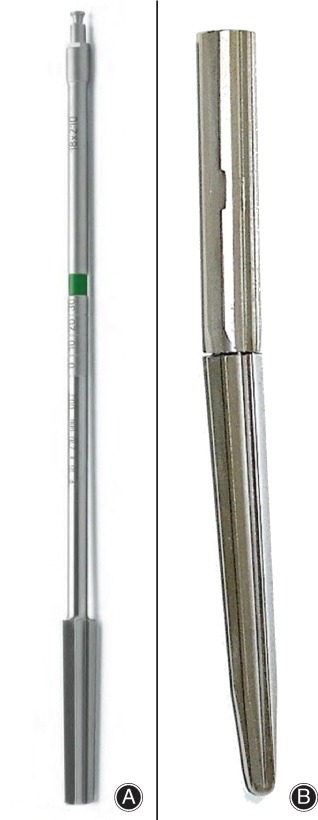
Different reamer designs for preparation of the femoral canal using (A) a conical straight reamer or (B) a conical kinked reamer.

We investigated 10 synthetic femoral bones (Model #3403 medium left, generation 4, composite, from the company Sawbone, Vashon, Washington, USA). The standard measurements included a caput–collum–diaphyseal (CCD) angle of 135°, a midshaft outer diameter of 27 mm, and an inner canal diameter of 13 mm. Prior to implantation of the stem into the synthetic bones, femora for the two groups were prepared by sawing the proximal (subtrochanteric) portion. For accurate intramedullary interfaces between the bone and the fluting part of the stem, the resection line was based on the nominal length and diameter of the stem. The inner canals of the femora were reamed to match the stem diameter, which was performed by following the operation instructions that had been provided with the equipment. In the first group, the intramedullary canal preparation was performed with a straight conical reamer (SR group, Fig. 3A). The femoral preparation of the second group was conducted with a kinked conical reamer (KR group) with 3° of angulation, which was a prototype of the LINK prosthesis (Fig. 3B). Subsequently, the kinked stem was inserted into the femora using an endofemoral procedure. Strict attention was paid to restoration of the original length of the femur and to implantation of the stem to a sufficient depth.

### *Metal‐bone Interface Analysis*

**Figure 4:**
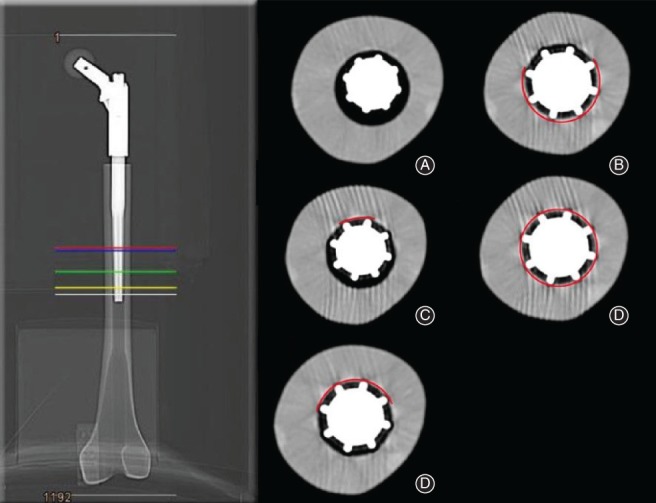
Analysis of the metal–bone interface with cross‐section references of the stem using a CT scan; (A) white line with 0% stem–bone interface, (B) yellow line with 25% stem–bone interface, (C) green line with 50% stem–bone interface, (D) blue line with 75% stem–bone interface and (E) red line with 100% stem–bone interface.

The specimens were analyzed using a CT scanner (BrightSpeed Performix 16 SI, General Electronic Healthcare Germany, Munich, Germany) to evaluate the metal–bone interface (Fig. 4A–E). The scan thickness was 0.625 mm. The proportion of contact between the implant and the inner cortical bone in each image was determined as the percent value of the implant circumference: for example, 0%, 25%, 50%, 75% or 100%. A value of 100% corresponds to contact of the entire circumference between the implant and cortical bone; 0% indicates that the implant was positioned in the medullary cavity without any contact with cortical bone. To determine the anchored implant length in millimeters in each case, the abovementioned image classifications (0% to 100%) were multiplied by the slice thickness. In addition, the total length of the stem–bone–interface (mm) and the 100% metal–bone–contact in relation to the total interface (%) were determined. We evaluated these data using the AGFA IMPAX EE CD Viewer program (Agfa Heath Care GmbH, Bonn, Germany).

### *Mechanical Testing System*

The experiments were conducted with a servohydraulic testing device (Instron 8874 Table‐Model Axial‐Torsion Fatigue Testing Systems, Instron Germany GmbH, Darmstadt, Germany). For the load cell, the axial force capacity was ±10 kN, the torque capacity was ±100 Nm, and the accuracy was ±0.5%. The test reports were all grouped automatically by the FastTrack console software used by the multi‐axis fatigue system (FTStartUp V. 7.22, MAX V. 9.2, Instron). We ensured that all hardware devices were working and that the same devices were used for all specimens.

### *Axial Stiffness Tests*

**Figure 5:**
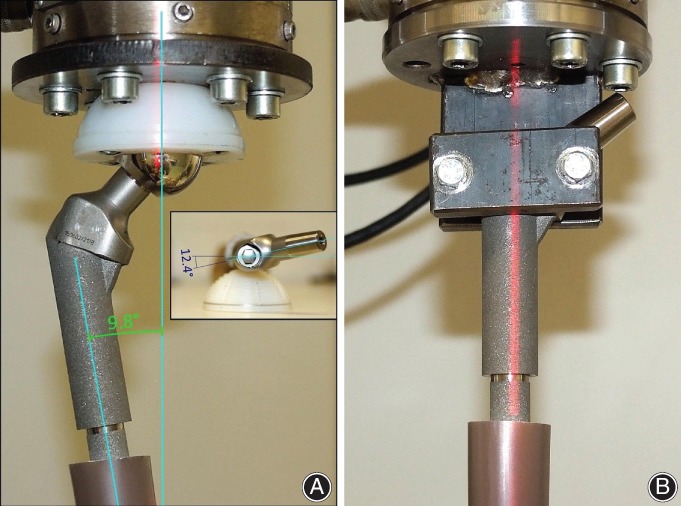
A specimen that is clamped in the servohydraulic testing device for calculation of (A) the axial stiffness with 10° adduction and (B) the torsional stiffness.

To precisely analyze the axial stiffness of the specimen, biomechanical conditions were simulated. We arranged the axes of the femora in a 10° movement towards the median line due to the force transmission in the proximal femur, simulating the single‐legged phase of walking. Depending on the specimen, the condyle region was fixed in a negative imprint in a vice. Then, the stem end was inserted into a suitable femur head. By fixating a compatible inlay on a metal plate of the testing device, the forces were transmitted to the specimen. Vertical loads with a linear ramp‐up/ramp‐down waveform consisting of 10 000 cycles and a load of 2.4 kN were applied to the femur head (see Fig. 5A). For the simulation, the load of a normal gait cycle force was transferred by a two‐phase wave form onto the specimen.

### *Torsional Stiffness Tests*

To enable testing of the torsional stiffness, the long axes of the femora were aligned vertically. This test was performed in the frontal and sagittal planes using a leveling gage, and the positioning was maintained in relation to that of the loading machine. The end of the stem was attached to a metal plate with a screw. Torsional loads were then applied using a linear ramp‐rotation (10 000 cycles with 10 Nm, axial pre‐load of 0.8 kN, as shown in Fig. 5B).

### *Statistical Analysis*

For the statistical analysis, a two‐sided Mann–Whitney *U*‐test was used to detect group differences for each parameter. This included the axial and torsional stiffness, the total stem migration, and the contact area between stem and bone. A correlation between the contact area and the stem migration was tested by calculating the Pearson correlation coefficient. All statistical tests were carried out at a significance level of *P* < 0.05 using the Software XLSTAT (Addinsoft version 19.7, Paris, France).

## Results

### *Metal–bone Interface*

Each sample was scanned and inspected, and the femur–stem interface areas were then calculated. No significant differences were observed among the contact patterns of the implants against the inner cortical bone (Table 1).

**Table 1:** Metal–bone interface lengths

Contact area	Straight reaming (mean ± SD)	Kinked reaming (mean ± SD)	*P*‐value
25% contact (mm)	14.75 ± 5.26	10.93 ± 1.83	n.s.
50% contact (mm)	11.25 ± 5.06	10.53 ± 4.42	n.s.
75% contact (mm)	12.88 ± 11.12	7.57 ± 3.25	n.s.
100% contact (mm)	47.38 ± 10.23	50.73 ± 4.17	n.s.
Total interface (mm)	86.25 ± 1.25	79.63 ± 4.70	n.s.
100% contact in relation to total interface (%)	54.97 ± 12.02	63.82 ± 6.87	<0.05

n.s., not significant.

Implants with a straight conical reaming preparation corresponded well with the conical anchorage system, and the cortical contact circumference was more than half of the total anchorage length. In contrast, the group with kinked intramedullary femoral preparation exhibited conical surface contacts along nearly two‐thirds of the anchoring track (*P* > 0.05). No differences were found between the two groups for local resolution of implant impaction. Despite different intramedullary preparations, the SR group demonstrated conical and multiple‐point anchorage, and the KR group exhibited more conical surface anchorage.

### *Stem Migration*

**Figure 6:**
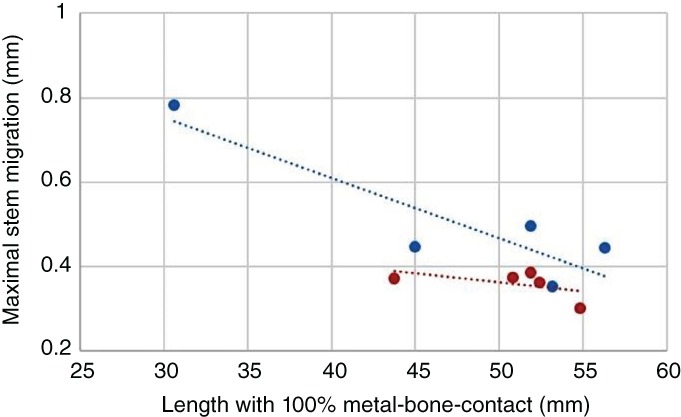
Correlation between the absolute stem migration and the anchoring length with 100% metal–bone contact (blue, straight reaming; red, kinked reaming).

The migration patterns of both groups were practically identical but without an asymptotic relationship, so that, at least in a first approximation, they could be assessed in a logarithmic manner. Among the various specimens, the absolute migration distances from the first to the ten‐thousandth gait cycle were apparently the same (SR group 0.51 ± 0.16 mm *vs* KR group 0.36 ± 0.03 mm, *P* = 0.095). For the SR group, a correlation was found between the completely conical anchorage length and absolute migration (*P* < 0.05, *R* = 0.89; Fig. 6). Longer conical anchorage resulted in less migration of the implant. After kinked intramedullary femoral preparation, this correlation disappeared, but this group showed decreased maximal stem migration, resulting in equally good results to those in the SR group.

### *Torsional Stiffness*

Torsional loading does not cause any implant migration, but this is not the case for axial loading. Therefore, for all of the implants under investigation, the torsional stiffness remained unchanged from the first to the last gait cycle, and no differences were observed between the study groups (SR group 6.48 ± 0.17 Nm/° *vs* KR group 6.52 ± 0.25 Nm/°, *P* = 0.398).

### *Axial Stiffness*

**Figure 7:**
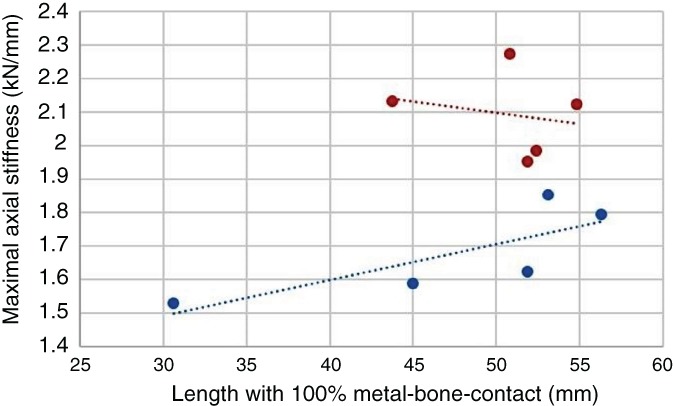
Correlation between the absolute axial stiffness and the anchoring length with 100% metal–bone contact (blue, straight reaming; red, kinked reaming).

Due to migration in all samples, an increased axial stiffness was observed during the gait cycles, but this increase did not lead to an asymptotic relationship. The maximum axial stiffness after 10 000 cycles differed significantly between the 2 groups (SR group 1.68 ± 0.14 kN/mm *vs* KR group 2.09 ± 0.13 kN/mm, *P* = 0.008, Fig. 7). Furthermore, the group with kinked stems and a kinked femoral preparation showed less deviation than the SR group. No correlation was observed between the anchoring length and 100% metal–bone contact in either group.

## Discussion

The current biomechanical investigation found that the maximal stem migration of a kinked stem was correlated with the circumferential conical anchoring length after straight reaming of the proximal femoral canal. Conversely, the kinked implant showed a significantly higher maximal axial stiffness after preparation of the femoral intramedullary canal with a kinked reamer. After 10 000 simulated gait cycles, it was 24% higher, indicating a better anchorage of the stem. The torsional stiffness levels of the specimens were equivalent for the two different reaming groups and did not change over the simulated gait cycles. The cross‐sectional stem design might, therefore, determine the rotational stability more than the type of stem anchorage in the coronal and sagittal plane.

Many years have passed since cementless revision arthroplasty with distal fixation was first shown to be a successful method. This technique involves the principles of press‐fit and multiple‐point impaction^21^, ^22^, ^23^, ^24^, ^25^. Press‐fit anchorage itself is based on conical impaction of the implanted stem onto the prosthesis bed of a femur that has previously been prepared. Multiple‐point impaction results from the incongruence of stem design and femur form, which leads to contact between the inner cortical bone and the stem at no fewer than three points. The press‐fit procedure is the method of choice to achieve primary stability of femoral revision prostheses, resulting in secondary stability due to increased implant osteointegration. In this context, some investigations have shown that the surgical approach could influence this anchoring principle. As shown in cadaver studies by Fink *et al*., implantation of a curved stem in a circular area fixation could be accomplished only after a transfemoral procedure was used, which is consistent with a press‐fit fixation of a straight stem^7^, ^10^. It was also demonstrated that in the case of endofemoral implantation of a curved stem, only three‐area fixation occurs, and a longer and thinner implant is required for sufficient anchorage. Completely different results were observed when more extensive defects of the proximal femur were indicated. For example, no significant differences could be observed regarding the parameters of stem migration or axial and torsional stiffness after endofemoral implantation of the two stem designs (straight *vs* kinked)^19^. The authors argued that if the best possible preparation with a straight conical reamer is achieved, a combination of conical and 3‐point impaction always occurs, regardless of the stem design. Biomechanical analysis of revision total knee arthroplasty showed that the mechanical stability of cementless press‐fit stems may be enhanced by optimal reaming, which affects the stem length inserted and the radial interference^26^, ^27^. Ferguson *et al*. found that the torsional stiffness was increased 2.2 times for cylindrical versus flexible reamers in cases with the same type of cementless press‐fit stem^28^. Furthermore, over‐reaming may increase the stem insertion length, and under‐reaming may increase the metal–bone interface, which improves mechanical stability. For these situations, valid hypotheses have suggested that the prepared prosthesis bed of the femur influences the anchoring principles of cementless revision stems.

For evaluation of this issue, a kinked stem was chosen in this study. This implant is a compromise between the non‐anatomic straight stem and the anatomic curved stem. The kinked stem follows the antecurvation of the femur and allows customized preparation of the femur with a kinked reamer. This conical reamer prototype with a kinked geometry of 3° of angulation is intended to better align with the curvature of the femur than that of a straight conical reamer. In the investigated osseous defect model, the different intramedullary preparations of the proximal femur did not differ in localization or length of fixation of the kinked stem. Conversely, a correlation between the maximal stem migration after straight reaming and the circumferential conical anchoring length was found. The bigger the area of conical stem anchorage, the better was the primary stability of the stem. Femoral preparation with a kinked reamer showed no correlation with the circumferential conical anchoring length, but equivalent results were found for the maximal migration, with less deviation than the straight reamer. It could be assumed that the kinked stem is supported flatly by the 3° angulation after kinked reaming, resulting in a higher primary stability. No differences were found with regard to torsional stiffness or the correlation with the amount of metal–bone interface between the two groups. The torsional stiffness is generated by the star profile of the tested implants. It can be assumed that a short circumferential anchoring length is sufficient to achieve maximal torsional stiffness. In addition, the results showed that the axial stiffness of both groups was completely independent of the conical anchorage length. It is notable that the maximal axial stiffness of the tested stems was significantly increased, with less deviation in the group with kinked reaming than that with straight reaming.

Our study has several limitations. The biomechanical stability of the press‐fit stem and the comparison associated with it in each group depended on equal reaming of the synthetic femur. Reaming is associated with the intramedullary stem length and with the impaction pressure of the prosthesis on radial interference. The main principle of fixation also involves the cortical force distribution and yields an individual press‐fit result. In our opinion, the most crucial limiting factor was adapting a piece of synthetic bone to simulate actual *in vivo* conditions. For revision arthroplasty (e.g. with respect to the amount of bone stock, interfacial friction, long‐term influence of bone ingrowth, axial deviations, and periprosthetic fractures, etc.), the qualities of a synthetic femur are quite different from those of a real human femur, especially when different reamer designs are compared. In conclusion, the implantation of a kinked stem after kinked conical intramedullary preparation of the proximal femur showed equivalent results regarding anchoring length, stem migration, and torsional stiffness to straight conical reaming preparation. In terms of axial stiffness, the specimens with kinked reaming showed significantly higher values. Prospectively, we will advocate the use of a kinked reamer system for the implantation of kinked cementless stems for revision hip arthroplasty.
